# A novel scoring system for early assessment of the risk of the COVID-19-associated mortality in hospitalized patients: COVID-19 BURDEN

**DOI:** 10.1186/s40001-022-00908-4

**Published:** 2023-01-03

**Authors:** Mohammad Hossein Imanieh, Fatemeh Amirzadehfard, Sina Zoghi, Faezeh Sehatpour, Peyman Jafari, Hamidreza Hassanipour, Maryam Feili, Maryam Mollaie, Pardis Bostanian, Samrad Mehrabi, Reyhaneh Dashtianeh, Afrooz Feili

**Affiliations:** 1 Gastroenterohepatology Research Center, Shiraz University of Medical Sciences, 9th Floor, Mohammad Rasoul Allah Research Tower, Khalili St, PO Box: 7193635899, Shiraz, Iran; 2 Sleep Disorders Laboratory, Namazi Hospital, Shiraz University of Medical Sciences, Shiraz, Iran; 3 Division of Pulmonology, Department of Internal Medicine, School of Medicine, Shiraz University of Medical Sciences, Shiraz, Iran; 4 Department of Internal Medicine, Shiraz University of Medical Sciences, Shiraz, Iran; 5 Department of Biostatistics, Shiraz University of Medical Sciences, Shiraz, Iran; 6 Student Research Committee, Shiraz University of Medical Sciences, Shiraz, Iran

**Keywords:** COVID-19, SARS-CoV-2, Mortality, Prognosis, Risk assessment

## Abstract

**Background:**

Corona Virus Disease 2019 (COVID-19) presentations range from those similar to the common flu to severe pneumonia resulting in hospitalization with significant morbidity and/or mortality. In this study, we made an attempt to develop a predictive scoring model to improve the early detection of high risk COVID-19 patients by analyzing the clinical features and laboratory data available on admission.

**Methods:**

We retrospectively included 480 consecutive adult patients, aged 21–95, who were admitted to Faghihi Teaching Hospital. Clinical and laboratory features were collected from the medical records and analyzed using multiple logistic regression analysis. The final data analysis was utilized to develop a simple scoring model for the early prediction of mortality in COVID-19 patients. The score given to each associated factor was based on the coefficients of the regression analyses.

**Results:**

A novel mortality risk score (COVID-19 BURDEN) was derived, incorporating risk factors identified in this cohort. CRP (> 73.1 mg/L), O_2_ saturation variation (greater than 90%, 84–90%, and less than 84%), increased PT (> 16.2 s), diastolic blood pressure (≤ 75 mmHg), BUN (> 23 mg/dL), and raised LDH (> 731 U/L) were the features constituting the scoring system. The patients are triaged to the groups of low- (score < 4) and high-risk (score ≥ 4) groups. The area under the curve, sensitivity, and specificity for predicting mortality in patients with a score of ≥ 4 were 0.831, 78.12%, and 70.95%, respectively.

**Conclusions:**

Using this scoring system in COVID-19 patients, the patients with a higher risk of mortality can be identified which will help to reduce hospital care costs and improve its quality and outcome.

**Supplementary Information:**

The online version contains supplementary material available at 10.1186/s40001-022-00908-4.

## Introduction

Corona Virus Disease 2019 (COVID-19) caused by the Severe Acute Respiratory Syndrome Coronavirus-2 (SARS-CoV-2) is highly a contagious disease, with symptoms ranging from those of common flu including fever, cough, and congestion of the nasal cavity to very severe respiratory symptoms [1]. As the pandemic spread, other symptoms such as loss of taste and smell (anosmia) have also emerged [2, 3]. Patients with the severe form of the disease can experience a large range of symptoms arising from the host immune response against the infection including serious respiratory disease and pneumonia, vascular and hemodynamic disorders, and metabolic dysfunction [4–7]. Patients can also present normal or abnormal leukocyte counts, lymphopenia, or thrombocytopenia, with extended activated thromboplastin time, and elevated C-reactive protein (CRP) [5, 8, 9]. Those most at risk are the elderly and people with preexisting medical conditions, such as cardiovascular disorders and diabetes mellitus [10–12]. The exact mechanisms behind the disease and why some remain asymptomatic carriers, while other patients develop severe diseases with unfavorable outcomes are still poorly understood [13].

Here, we present details of the patients with laboratory-confirmed COVID-19 pneumonia to shed light on the specifications of patients who experienced in-hospital mortality and explore the risk factors that might facilitate early screening. Finally, we develop a predictive model for early detection of COVID-19 patients with a high risk of mortality using analysis of clinical features and laboratory data on admission, allowing timely identification and intervention.

## Materials and methods

We retrospectively investigated 480 adult patients, aged 21–95, who were admitted to Faghihi Teaching Hospital from September 23, 2020, to November 21, 2020, with a positive RT-PCR COVID-19 test. The patients who did not complete their course of hospitalization and were released with their own consent were excluded. The study protocol was approved by the Ethics Committee of Shiraz University of Medical Sciences (IR.SUMS.MED.REC.1400.382). Written informed consent was obtained from all participants on admission.

Corresponding medical records were thoroughly examined. Variables investigated in this study for correlation with the outcome of hospitalization (discharge or death) included demographics (gender and age), previous medical conditions (smoking/opium abuse, hypertension, HTN, and diabetes mellitus, DM, ischemic heart disease, IHD, and hyperlipidemia, and HLP), previous drug history (angiotensin-converting-enzyme inhibitors/angiotensin II receptor blockers; ACEIs/ARBs, calcium channel blocker; CCB, beta blockers; BB, acetylsalicylic acid; ASA, and Statins) the patients’ condition on admission (systolic blood pressure; SBP, diastolic blood pressure; DBP, pulse rate; PR, respiratory rate; RR, temperature; T, O_2_ saturation; O_2_ Sat, presence of dyspnea, cough, chest pain, fever, malaise, anorexia, nausea and/or vomiting; N/V, other gastrointestinal symptoms, and the interval between disease onset and hospital admission), the laboratory findings of the sample obtained on admission (white blood cell count; WBC, absolute neutrophil count; ANC, absolute lymphocyte count; ALC, hemoglobin; Hb, prothrombin time; PT, partial thromboplastin time; PTT, blood urea nitrogen; BUN, creatinine; Cr, sodium; Na, potassium; K, aspartate transaminase; AST, alanine transaminase; ALT, alkaline phosphatase; ALP, albumin; Alb, total bilirubin; TB, direct bilirubin; DB, creatinine phosphokinase; CPK, lactate dehydrogenase; LDH, erythrocyte sedimentation rate; ESR, C-reactive protein; CRP), and total length of stay in the hospital.

### Statistical analysis

Quantitative variables are presented as mean ± SD and qualitative variables as frequency (percentage) for all independent variables. Univariate analysis was applied to identify the potential risk factors of mortality using independent *t*‐test and chi-square test as appropriate. Statistically significant variables (significance level set at 0.05) that were of concern from a clinical perspective were subsequently employed in multiple logistic regression analysis with backward elimination method to identify the predictive factors. To formulate and obtain a clinical predictive risk score, we followed a similar strategy used by Ho et al. [14]. Accordingly, the continuous prognostic variables that were significant in the univariate analysis were categorized using the receiver operating characteristic (ROC) curve. Then, all significant categorical variables in the univariate analysis were re‐entered into a logistic regression model. According to the relative contribution of each variable in the logistic regression model, which was determined by the regression coefficient, an integer score was assigned to each categorical variable. Finally, the scores were organized to obtain a practical triage of the patients into low‐ and high‐risk cases. SPSS version 23 (SPSS Inc, IBM, New York, NY) and MedCalc statistical program, version 19.5 (MedCalc Software, Mariakerke, Belgium) were used to analyze the data. This study was conducted and written in accordance with the TRIPOD Statement.

## Results

### Patient details and hospitalization

A total of 1511 patients were visited at the triage of Faghihi hospital with the impressions related to COVID-19, of whom 480 patients (212 females and 268 males) fulfilled the criteria set by this study. The median age of the patients included was 61 years old (IQR: 49–72). The median days from the onset of symptoms to admission was 8 ± 6 days, and the total length of stay in the hospital was 5 days (IQR: 3–9). Intensive care unit (ICU) admission of COVID-19 patients is significantly associated with the worse outcome of hospitalization (*P* value < 0.0001).

### Comparison of the associated factors

The patients were assigned into two groups (discharged = 312 and expired = 168). In total, 48 demographic, clinical, and laboratory variables were compared in both groups according to the outcome of hospitalization to determine meaningful ones (Additional file 1: Table S1). Multiple logistic regression analysis was carried out separately to assess the effect of 19 variables identified as significant independent predictors of outcome in our cohort (Table 1).

**Table 1:** Demographic, clinical, and laboratory characteristics of COVID-19 patients significantly associated with mortality, stratified by the outcome of hospitalization

Variable	Discharged (*n* = 312)	Expired (*n* = 168)	*P value*	Cutoff set for ratio variables
Age Mean (SD)	57.03 (15.759)	66.80 (13.930)	< *0.0001*	< 60
				> = 60
HTN (present:absent)	115:197	80:88	*0.025*	–
IHD (present:absent)	43:269	40:128	*0.008*	–
N/V (present:absent)	75:236	27:140	*0.047*	–
Systolic BP	127.97 (19.083)	123.05 (24.884)	*0.026*	> 114
				< = 114
Diastolic BP	79.44 (11.350)	75.06 (13.180)	*0.001*	> 75
				< = 75
RR	20.50 (4.715)	23.86 (7.436)	< *0.0001*	< = 20
				> 20
O_2_ Sat	84.63 (10.539)	70.42 (16.926)	< *0.0001*	> 90
				83–90
				< = 83
WBC	7.7684 (4.17933)	10.1776 (5.87533)	< *0.0001*	< = 7.52
				> 7.52
ANC	6045.06 (3850.1621)	8573.50 (5173.281)	< *0.0001*	> 5628
				< = 5627
PT	32.92 (23.700)	42.31 (26.991)	< *0.0001*	< = 16.2
				> 16.2
BUN	21.16 (26.450)	34.76 (24.786)	< *0.0001*	< = 23
				> 23
Cr	1.4259 (1.46007)	1.9012 (1.81179)	*0.002*	< = 1.3
				> 1.3
K	4.452 (0.5787)	4.811 (0.8522)	< *0.0001*	< = 4.9
				> 4.9
AST	67.74 (99.519)	116.94 (305.125)	0.015	< = 67
				> 67
Alb	3.748 (0.3469)	3.446 (0.4644)	< *0.0001*	> 3.7
				< = 3.7
CPK	218.35 (312.488)	634.76 (1432.796)	< *0.0001*	< = 78
				> 78
LDH	742.83 (561.077)	1055.28 (637.258)	< *0.0001*	< = 731
				> 731
CRP	64.39 (50.420)	72.99 (21.225)	0.049	< = 73.1
				> 73.1

These variables entered multivariate logistic regression analysis. *HTN* hypertension, *IHD* ischemic heart disease, *N/V* nausea and/or vomiting, *BP* blood pressure, *RR* respiratory rate, *O2 Sat* O2 saturation, *WBC* white blood cell count, *ANC* absolute neutrophil count, *PT* prothrombin time, *BUN* blood urea nitrogen, *Cr* creatinine, *K* potassium, *AST* aspartate transaminase, *Alb* albumin, *CPK* creatinine phosphokinase, *LDH* lactate dehydrogenase, *CRP* C-reactive protein

To generate a scoring system, O_2_ Sat, DBP, PT, BUN, LDH, and CRP values, among other continuous variables, were categorized using integer cut-points guided by the receiver–operator characteristic (ROC) curve.

### Final model and COVID-19 BURDEN risk score

To formulate a numerical scoring model, we used the coefficients generated by the logistic regression equation, to create an integer number, approximating the values of the coefficients for each of the categories in Table 2.

**Table 2:** Variables, which remained in the final multiple logistic regression model to predict the risk of mortality during hospitalization

Variables	Coefficient	Coefficient (SE)	*P value*	Odds ratio (95% CI)	Lower –Upper 95% CI
DBP	0.794	0.360	*0.027*	2.212	1.093–4.479
PT	0.761	0.349	*0.029*	2.140	1.079–4.242
BUN	1.254	0.356	< *0.0001*	3.503	1.744–7.039
LDH	1.066	0.369	*0.004*	2.903	1.408–5.987
CRP	1.017	0.418	*0.015*	2.766	1.220–6.271
84% ≤ O2 Sat ≤ 90%	0.799	0.652	< *0.0001*	2.223	0.620–7.975
O2 Sat < 84%	2.218	0.604	< *0.0001*	9.189	2.811–30.040

O2 Sat > 90% was used as reference index in O2 saturation category. Formulation of integer risk score for each category was based on the strength of contribution to logistic equation based on the coefficient (for example, the coefficient of O2 Sat < 84% is 2.218; therefore, an integer score of 2 was given. Coefficient of BUN is 1.254; therefore, an integer score of 1 is given)

*DBP* diastolic blood pressure, *PT* prothrombin time, *BUN* blood urea nitrogen, *LDH* lactate dehydrogenase, *CRP* C-reactive protein, *O2 Sat* O2 saturation

The Italic was merely used to show the elevate the p values above other values and make a distinction

Using the coefficients of the regression analyses, the O_2_ Sat greater than 90%, 84–90%, and less than 84% were given scores of 0, 1, and 2, respectively. Scores of either 0 or 1 were given to other variables including CRP, PT, DBP, BUN, and LDH levels (Table 3).

**Table 3:** Integer risk score attributable to each category derived from the coefficients of the logistic regression equation

Variables	Points awarded
C-reactive protein (CRP) mg/L	
≤ 73.1 mg/L	0
> 73.1 mg/L	1
O_2_ saturation variation (O_2_ sat Variation)	
Greater than 90%	0
84–90%	1
Less than 84%	2
Increased prothrombin time (increased PT)	
≤ 16.2 s	0
> 16.2 s	1
Diastolic blood pressure (DBP) mmHg	
> 75 mmHg	0
≤ 75 mmHg	1
Blood urea nitrogen (BUN) mg/dL	
≤ 23 mg/dL	0
> 23 mg/dL	1
Raised lactate dehydrogenase (Raised LDH) (U/L)	
≤ 731 Units/lit (U/L)	0
> 731 Units/lit (U/L)	1

COVID-19 BURDEN risk score = (CRP score) + (O_2_ sat Variation score) + (Increased PT score) + (DBP score) + (BUN score) + (Raised LDH score). The overall COVID-19 BURDEN risk score is between 0 and 7; the higher the score, the higher the risk of mortality during the course of hospitalization. The cutoff of the risk factor is set at 4. The patients with a score < 4 are categorized as low-risk, having a more favorable outcome, while those with a score ≥ 4 were more likely to have an undesirable outcome

A novel mortality risk score (termed COVID-19 BURDEN) was derived, incorporating risk factors identified in this cohort. COVID-19 BURDEN is an acronym for CRP (> 73.1 mg/L), O_2_ saturation Variation (greater than 90%, 84–90%, and less than 84%), Increased PT (> 16.2 s), Diastolic blood pressure (≤ 75 mmHg), BUN (> 23 mg/dL), and Raised LDH (> 731 U/L), as shown in Table 3.

Using the new scoring system, we scored each patient and compared him/her with his/her eventual outcomes. For an individual patient, the total score was derived from the sum of the score attributed to the variables mentioned above. The minimum and maximum possible scores are 0 and 7, respectively.

**Fig. 1:**
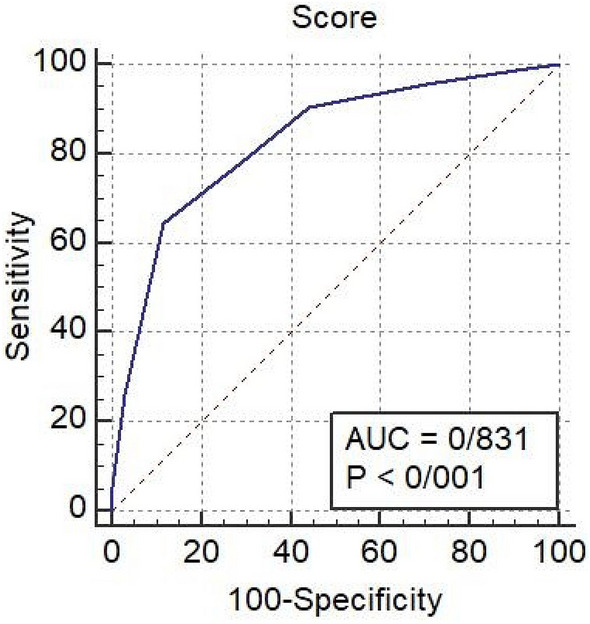
ROC curve demonstrates that the final fitted model has an adequate area under the ROC curve (AUC = 0.831, sensitivity = 78.12%, specificity = 70.95%)

It was possible to triage the patients to the groups of low- (score < 4) and high-risk (score ≥ 4) groups. ROC analysis for this risk score yielded an area under the curve (AUC) of 0.831 (AUC of 1.0 indicating a perfect test) (Fig. 1). The sensitivity and specificity of the model for predicting mortality in patients with scores of ≥ 4 were 78.12% and 70.95%, respectively.

## Discussion

The COVID-19 pandemic is a public health concern with dire health, environmental, and economic consequences [15]. Currently, the facilities required for the hospitalization of patients with COVID-19 are limited. This limitation is especially pronounced in ICUs for patients requiring mechanical ventilation [16]. It is necessary to develop prediction models that can be used by clinicians, to estimate the severity of the disease and prognosis of each patient at an early stage of the disease, which will grant a better allocation of resources and a wider window for interventions. This tool also potentially helps to reduce hospital care costs and improves its quality in the health care units [17–19]. Applying such prediction models is a useful strategy for the early screening of high-risk patients in crowded care centers during the COVID-19 outbreak. Risk prediction models are increasingly utilized in medical practice to help practitioners promote healthcare quality. Following extensive validations in independent samples, a predictive score can be used routinely.

Thus far, several prognostic models with varying clinical applicability and performance have been developed for COVID-19. Among them, a few have a low risk of bias and low concern for applicability. The most well-validated models are developed by Clift et al. and Knight et al. with the potential of being applied by clinicians in public health and clinical settings [30, 31]. Age, sex, RR, O_2_ sat, level of consciousness, BUN, BMI, and comorbidities are among the variables incorporated into the aforementioned models. On top of that, a few models developed before the pandemic were applied to COVID-19 prognostication. Most notably APACHE II had very promising results (area under the curve, 0.966) in predicting in-hospital mortality [32].

In this survey, we designed a simply calculated clinical risk score, using the patient's clinical characteristics and laboratory data, which can be used as a predictor to predict mortality, the most important outcome in the course of hospitalization and implicitly related to the severity of the disease, the need for hospitalization, and the possibility of ICU admission requirement during the hospitalization. Low DBP (≤ 75 mmHg), prolonged PT (> 16.2 s), increase in the level of LDH (> 731 U/L), BUN > 23 mg/dl, elevated CRP (> 73.1 mg/L), and decrease in oxygen saturation (< 84%) were identified as risk factors for disease severity among 480 adult patients.

Severe hypoxemia (O2 sat < 84%) had the highest odds ratio (OR = 9.19) among the 6 risk factors determining the mortality in our survey. This finding was consistent with a cohort study by Bahl et al. among 1461 patients in which O2 sat of ≤ 88% was associated with a higher mortality rate [20]. In the review conducted by Petrilli et al. among 4103 patients, oxygen saturation of < 88% (OR = 6.99) was also introduced as the most critical factor predicting the outcome of hospitalization on admission [21].

Recently, several studies have been conducted in different parts of the world to develop a simple scoring system to predict the prognosis and outcome of COVID-19 [19, 22–25]. The first scoring system to predict the severity of COVID-19, incorporating age, glomerular filtration rate (GFR), WBC, neutrophil count, and myoglobin, was developed by Zhang et al. in 2020 among 80 patients [22]. The low sample size was one of the limitations of their study. Moreover, the aforementioned model did not include any of the vital signs, which were shown to be quite important in determining the clinical course of COVID-19 patients. The findings of the study conducted by Altschul et al. are also in agreement with the present study. Old age (especially ≥ 80 years) mean arterial blood pressure ≤ 60 mmHg, O_2_ sat < 94%, BUN > 30 mg/dl, CRP > 10 mg/dl, and INR > 1.2 were identified as six risk factors that affect the mortality rate of COVID-19 patients. Their study was conducted in three major referral hospitals in New York City. However, the study data is limited to their urban population and may not be fully generalizable to other settings with different backgrounds [24]. In another study which was published in July 2020, old age, the presence of coronary heart disease, high procalcitonin, lymphopenia, and high d-Dimer level were associated with a high mortality rate in the affected population [25].

Having a precise, inexpensive, accessible, and straightforward prediction model will improve the patients' triage, and help to identify high-risk individuals, initiate timely interventions, and allocate resources efficiently [22, 26]. One of the advantages of our model is that it does not include lung high-resolution computed tomography (HRCT) scans, which contributes greatly to the health financial system during this pandemic. Rather, we used more accessible clinical and laboratory metrics to predict disease severity, which will reduce the costs, improve the timing of the decision-making in the initial screening, and lift the burden from the already exhausted imaging facilities. HRCT can be conducted later during the hospitalization of the patients rather than on admission in the high risk patients; however, the present study is not concerned with this issue and this hypothesis requires further investigation on the effect of applying prediction models on the burden imposed on various units in hospitals.

CRP, Oxygen saturation variation, increased PT, DBP, and BUN, and raised LDH (COVID-19 BURDEN) were detected as six factors for COVID-19 infection severity. Using this prediction model, the severity of the disease in the early stages of the disease can be estimated and helps to reduce health costs and improve the quality of patient care in the health care units. The great utility of clinical prediction models have been demonstrated previously in several instances (e.g., CURB65, MELD, etc.) [27, 28].

The present survey was mainly limited by studying COVID-19 patients in a single center retrospectively. The management of the patients included in the present study was based on the most recent version of the WHO guideline for COVID-19 management at the time (https://www.who.int/publications/i/item/WHO-2019-nCoV-Clinical-2022.2) [29]. Furthermore, our model did not make use of viral load in predicting the mortality in COVID-19 patients. Viral load is among the most important parameters in the setting of COVID-19 infection; however, it is mostly limited to experimental settings and not implemented in the real-world clinical setting. Moreover, it is not easy-to-obtain and not available early in the admission of the patients, if at all. As a result, incorporating it into a screening model will defeat its purpose and make triage based on the model impossible. It is quite common for patients to receive non-invasive ventilation or high-flow nasal cannula before their screen either in the emergency department or during transportation with emergency medical service. In our study, although the clinicians conducting the primary screening abided by the recommendations for cessation of therapy before recording O_2_ saturation, it is plausible to assume that the O_2_ saturation was falsely recorded in a set of patients, inclining toward a higher O2 saturation. Undoubtedly, further studies with larger sample sizes are needed in settings with diverse backgrounds to validate the COVID-19 BURDEN model. Moreover, many more studies are still required to better understand the disease itself. Finally, simple models similar to that outlined in the present paper can be beneficial in other aspects of the care for COVID-19 patients and their needs including ICU admission requirements, unplanned readmission, its long-lasting health effects, etc.

## Supplementary Information

**Additional file 1: Table S1.** Demographic, clinical, and laboratory characteristics of COVID-19 patients, stratified by the outcome of hospitalization.

## Data Availability

All data generated or analyzed during this study are included in the final published article.
